# The removal of anionic and cationic dyes from an aqueous solution using biomass-based activated carbon

**DOI:** 10.1038/s41598-021-88084-z

**Published:** 2021-04-21

**Authors:** Nurul Umairah M. Nizam, Marlia M. Hanafiah, Ebrahim Mahmoudi, Azhar A. Halim, Abdul Wahab Mohammad

**Affiliations:** 1 Department of Earth Sciences and Environment, Faculty of Science and Technology, Universiti Kebangsaan Malaysia, 43600 Bangi, Selangor Malaysia; 2 Centre for Tropical Climate Change System, Institute of Climate Change, Universiti Kebangsaan Malaysia, 43600 Bangi, Selangor Malaysia; 3 Department of Chemical and Process Engineering, Universiti Kebangsaan Malaysia, 43600 Bangi, Selangor Malaysia; 4 Research Centre for Sustainable Process Technology (CESPRO), Faculty of Engineering and Built Environment, Universiti Kebangsaan Malaysia, 43600 Bangi, Selangor Malaysia

**Keywords:** Environmental sciences, Environmental chemistry

## Abstract

In this study, two biomass-based adsorbents were used as new precursors for optimizing synthesis conditions of a cost-effective powdered activated carbon (PAC). The PAC removed dyes from an aqueous solution using carbonization and activation by KOH, NaOH, and H_2_SO_4_. The optimum synthesis, activation temperature, time and impregnation ratio, removal rate, and uptake capacity were determined. The optimum PAC was analyzed and characterized using Fourier-transform infrared spectroscopy (FTIR), x-ray diffraction (XRD), a field emission scanning electron microscope (FESEM), Zeta potential, and Raman spectroscopy. Morphological studies showed single-layered planes with highly porous surfaces, especially PAC activated by NaOH and H_2_SO_4_. The results showed that the experimental data were well-fitted with a pseudo-second-order model. Based on Langmuir isotherm, the maximum adsorption capacity for removing methylene blue (MB) was 769.23 mg g^−1^ and 458.43 mg g^−1^ for congo red (CR). Based on the isotherm models, more than one mechanism was involved in the adsorption process, monolayer for the anionic dye and multilayer for the cationic dye. Elovich and intraparticle diffusion kinetic models showed that rubber seed shells (RSS) has higher α values with a greater tendency to adsorb dyes compared to rubber seed (RS). A thermodynamic study showed that both dyes’ adsorption process was spontaneous and exothermic due to the negative values of the enthalpy (ΔH) and Gibbs free energy (ΔG). The change in removal efficiency of adsorbent for regeneration study was observed in the seventh cycles, with a 3% decline in the CR and 2% decline in MB removal performance. This study showed that the presence of functional groups and active sites on the produced adsorbent (hydroxyl, alkoxy, carboxyl, and π − π) contributed to its considerable affinity for adsorption in dye removal. Therefore, the optimum PAC can serve as efficient and cost-effective adsorbents to remove dyes from industrial wastewater.

## Introduction

Achieving sustainable water management in an integrated and cross-cutting manner is one of the most significant challenges facing many countries. Sustainable water management includes a metric for wastewater treatment, which is vital in successfully managing wastewater and, ultimately, water quality. However, water contamination from different pollutants such as dyes has become a major environmental and health problem that poses a threat to society and living organisms^1,2^. The high concentration of dyes in natural water sources and industrial wastewater streams is a critical issue faced by many countries. The dyes are notorious for their persistence, high toxicity, and carcinogenic impurities. They also bioaccumulate in the food chain and, hence, the human body^3,4^. In recent studies, numerous toxic chemicals have been detected in drinking water at dangerous levels in many parts of the world^5–14^. The most common toxic pollutants are harmful chemicals and dyes in wastewater produced by heavy industries and other human activities that must be treated before discharged to the environment. Treatment must be accomplished using techniques that are robust, economically feasible, and environmentally friendly^15–20^.

Worldwide, environmental scientists’ focus has been developing efficient and sustainable technologies for water and wastewater management^2,21,22^. To overcome the shortcomings of more traditional approaches, cheaper and more efficient techniques to improve the quality of treated effluent have been proposed. Some of the widely-used methods include adsorption, membrane filtration, coagulation and flocculation, chemical precipitation, ion exchange, electrochemical removal, biosorption, reverse osmosis, and oxidation processes^7,17^^.^^23–28^. However, most of those methods involve high operational and maintenance costs. Among the techniques mentioned, the adsorption process using local biowastes is a cost-effective and efficient technique for removing toxic dyes and metal ions from wastewater^17^. Dyes are widely used in textile, food, printing, leather, and pharmacology industries^29–31^. The presence of dyes in aqueous solutions and the environment can affect the photosynthetic functions of plants in water by blocking the sunlight with its aromatic compounds and reducing dissolved oxygen^29,31–34^. Plus, some dyes, especially cationic and anionic, are also carcinogenic and mutagenic that can affect digestive tract irritation, skin irritation, and cyanosis^28–32^. In addition, cationic dyes like MB and anionic dyes such as CR are the most widely used dyes in industries^31^. Thus, it is crucial to control the release of these compounds to the environment.

The adsorption method typically uses activated carbon from natural resources that have high carbon content. Activated carbon is widely used to remove dyes from wastewater because of its convenience, ease of operation, simplicity of design, and reusability^1–3,35–37^. However, despite its extensive use in wastewater treatment, some commercial activated carbon remains an expensive material^35–37^. Therefore, interest in lower-cost alternatives to commercially available activated carbon that would still provide safe and economical methods of removing dyes from contaminated water has increased. For this reason, low-cost by-products of agriculture that able to act as an adsorbent have been recognized as a sustainable solution for wastewater treatment that minimizes waste, recovery, and reuse^6,15,17,21–24,38^. To support the sustainable option for removing dyes, PAC has been synthesized from rubber seed (RS) and its shell (RSS). These are one of the most common biomass wastes produced in Malaysia. RS and RSS are the waste by-products of agriculture and are generated consistently in bulk^39^. Thus, the synthesis of PAC from a different kind of biomass like RS and RSS has been extensively explored in the water treatment field. Therefore, PAC derived from RS and RSS can provide a high surface area and a specific affinity for the adsorption of dyes from aqueous systems^1,16,40–43^.

RSS has a smooth and impervious texture due to its chemical composition, influencing the PACs’ properties. Therefore, the study of possible effects of this biomass pretreatment may indicate the best way to produce PACs with specific physicochemical characteristics. Foo & Hameed^44^ reported that oxidative treatments (acids) and base treatments (alkaline) are excellent activation agents for activated carbon (AC) preparation. Oxidative treatment using acids can be associated with enhanced acidic functional groups (carboxylic, phenol, and carbonyl groups) on the AC surface. In contrast, basic treatment tends to have relatively large ion exchange capacities due to alkalinity and abundance of –OH groups. However, most of the related studies used only either one of the pretreatment methods mentioned^1,2,16,20,35,37,39–43,45^. To acknowledge the gap in this area, this study investigated the systematic effect of pretreatment using both acidic and basic activating agents to compare the characteristics using multiple characterization analyses.

This study focused on developing optimum operating conditions to synthesize PAC from RS and RSS for dye removal from aqueous solutions using carbonization, KOH, NaOH, and H_2_SO_4_ activation. The requirements for removal and uptake capacity, such as the effects of an adsorbent dose, pH, and time were studied. In addition, under optimum conditions, PAC was analyzed and characterized using physio-chemical techniques such as Fourier-transform infrared spectroscopy (FTIR), XRD, X-ray fluorescence (XRF), a FESEM, Zeta potential, and Raman spectroscopy. The operating conditions for the adsorption of dyes were optimized. In contrast, the studies of kinetics and isothermal behavior of adsorption and thermodynamic analysis were conducted under optimized conditions. The performance of the RS and RSS-derived adsorbents was compared. The results of those studies provided a better understanding of the mechanism for the adsorption of dye molecules onto the prepared PAC.

## Results and discussion

### Characterization of the synthesized PAC

**Figure 1:**
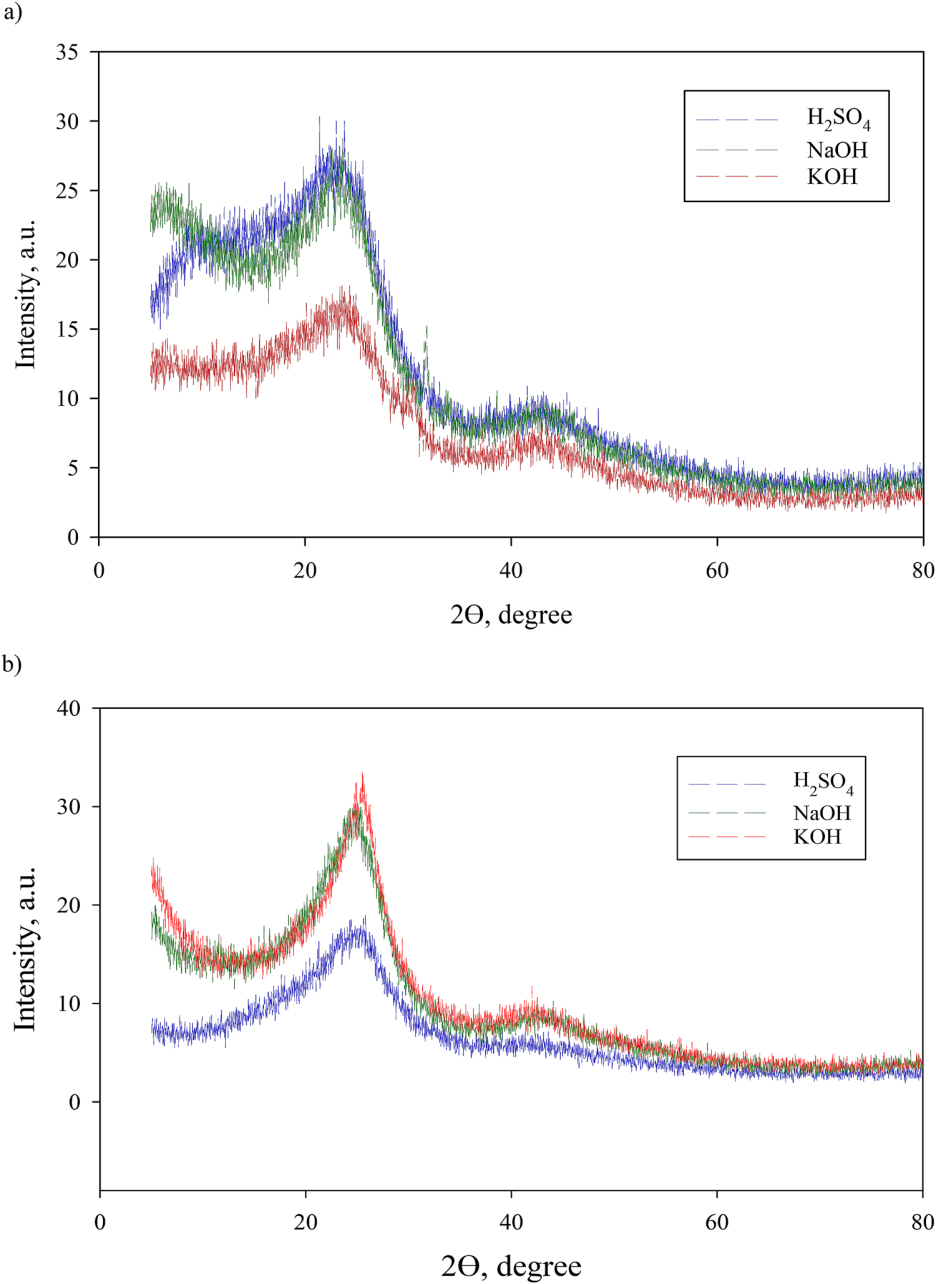
(**a**) XRD of the PAC from RSS based on activation solutions and (**b**) XRD of the PAC from RS based on activation solutions.

The XRD spectra of the PACs are shown in Fig. 1a–b. A broad diffraction peak of approximately 23.5° for all solutions corresponded to the activated carbon structure’s crystal plane. Spectra of RSS H_2_SO_4_ showed two strong peaks at 23.0° and 23.8°, one strong peak for NaOH, and no strong KOH peaks. In the spectrum of 45.1° of H_2_SO_4_, 45.0° of NaOH, and 44.5° of KOH, there were new and characteristic peaks, implying the existence of the PAC in metallic form. Based on the weakest peak and intensity shown, the KOH solution was the least effective among the three solutions for RSS, while H_2_SO_4_ and NaOH had almost the same effectiveness. However, RS’s spectra showed the total opposite where H_2_SO_4_ had only weak peaks, a diffraction peak for KOH sharper than others, followed by NaOH. The same characteristics were shown where these PACs exist in metallic form. Figure 1(b) revealed that the PAC might contain potassium compounds with high crystallinity after activation with KOH. This finding is similar to that for the tamarind seed-based AC^45^ and petroleum coked-based AC^46^ prepared with chemical activation.

**Figure 2:**
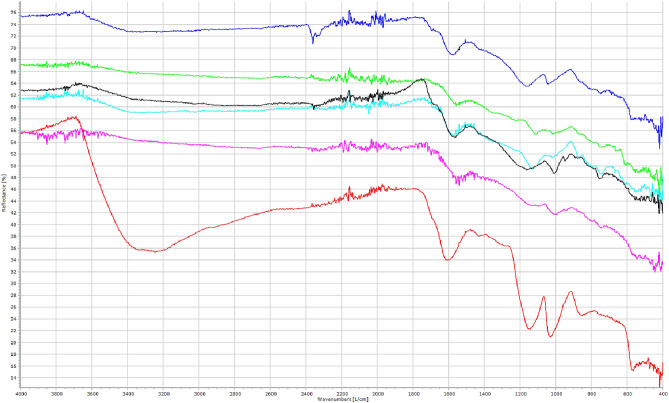
FTIR spectra of PAC adsorbent.

Figure 2 shows the spectra of the PAC adsorbents obtained using FTIR. The broad peaks characteristics of the vibration bands around 3851 cm^-1^ and 3,645 cm^-1^ corresponded to the O-H stretching of hydroxyl groups, while 3481 cm^-1^ indicated N-H stretching of the alkoxy groups^47–49^. The spectrum at 3300–3500 cm^-1^ was one of the most detected peaks in activated carbons obtained in previous studies^50,51^. In the PACs FTIR spectrum, the band centered around 2124 cm^-1^ corresponded to C=C stretching vibration and C=N bond. The bands around 1968 cm^-1^ and 1,593 cm^-1^ indicated C=O stretching and N–H bending vibrations of the alkoxy groups. The existence of those peaks demonstrated that the synthesized PACs contained abundant functional groups and proved the formation of hydrogen bonding between the functional groups, based on the slight shift of OH, C-O, and N-H functional groups.

**Figure 3:**
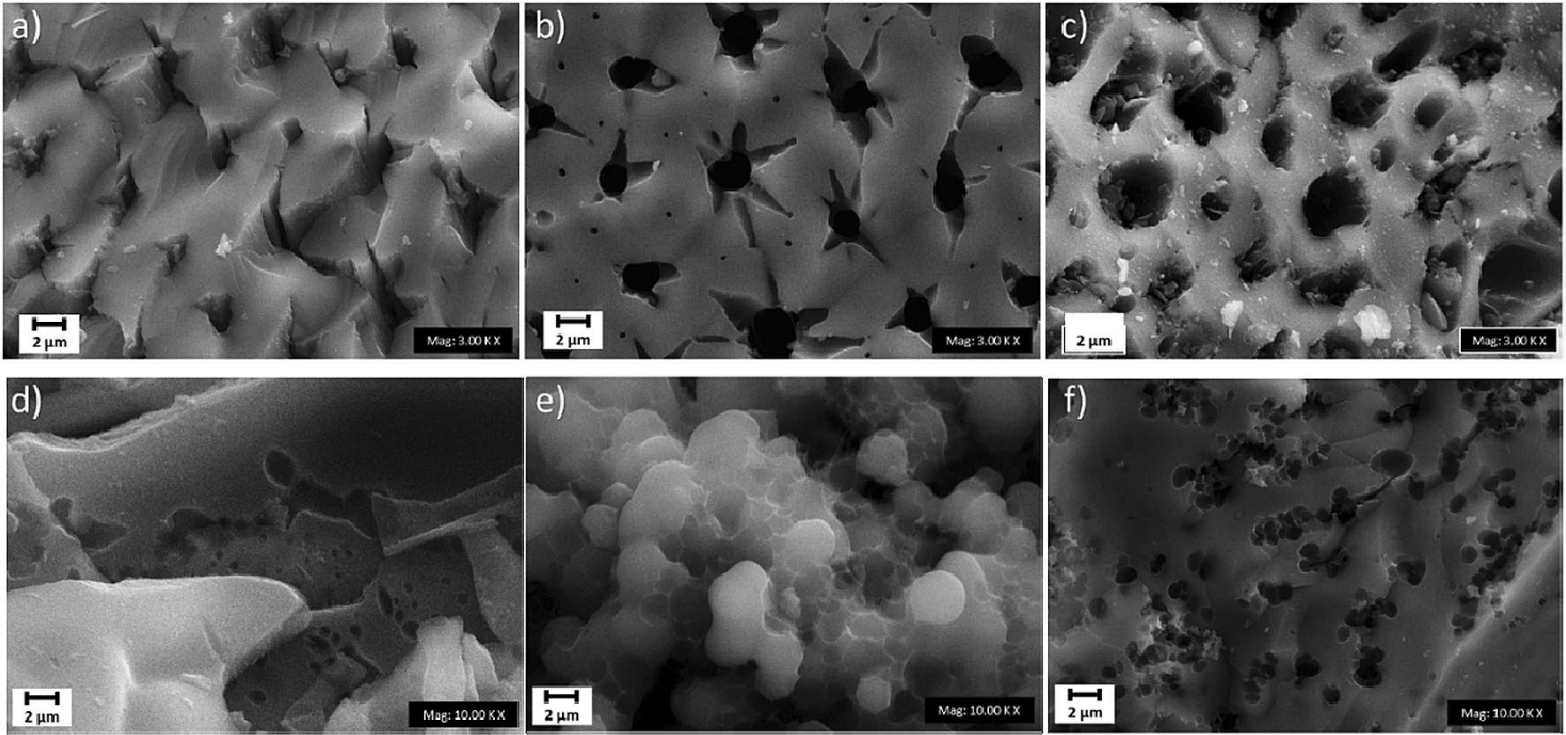
FESEM photographs of the PAC based on activation solutions of (**a**) RSS H_2_SO_4_, (**b**) RSS KOH, (**c**) RSS NaOH, (**d**) RS H_2_SO_4_, (**e**) RS KOH, and (**f**) RS NaOH.

**Figure 4:**
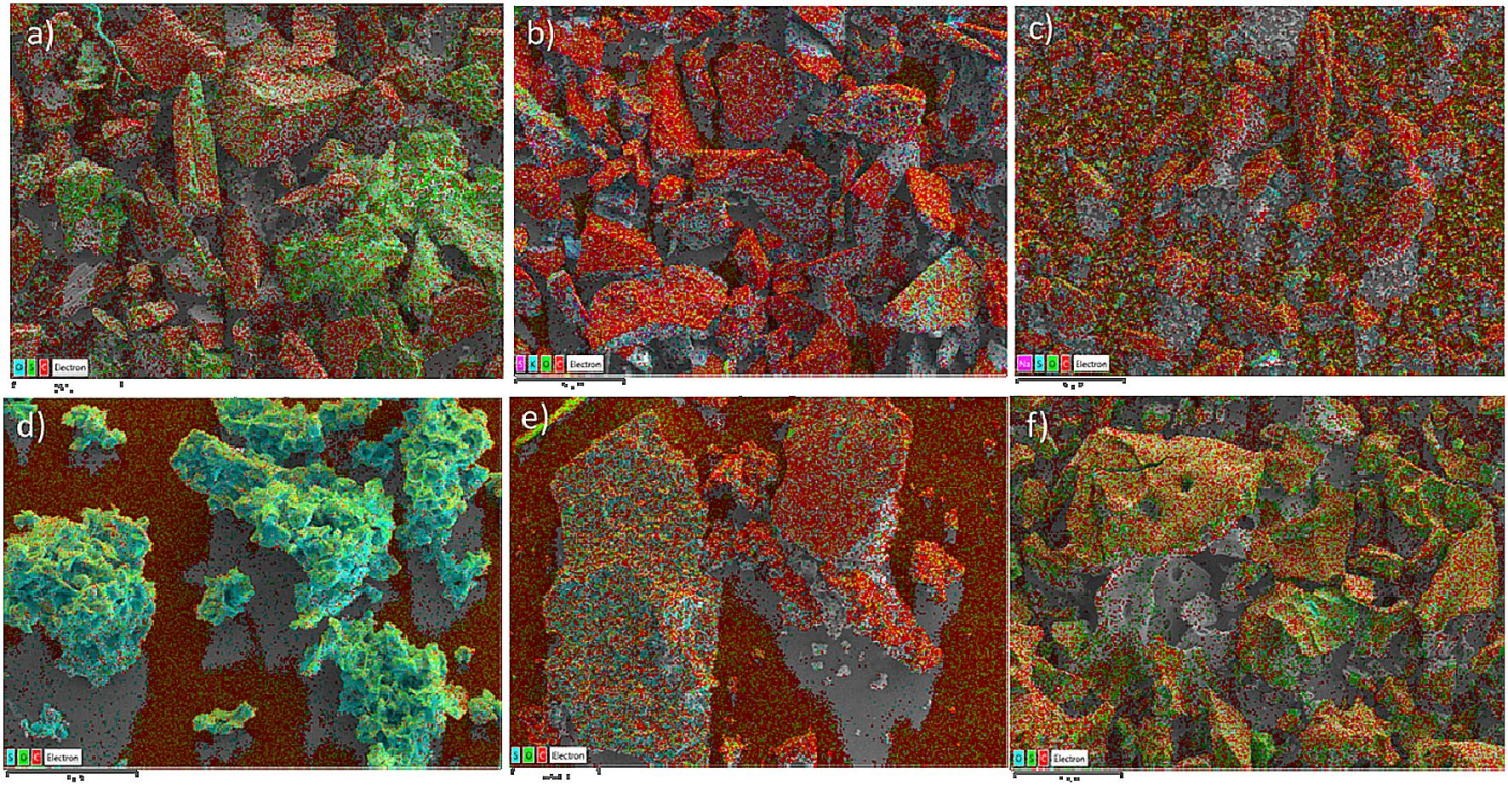
EDX mapping of the PACs based on activation solutions where (**a**) is RSS H_2_SO_4_, (**b**) is RSS KOH, (**c**) is RSS NaOH, (**d**) is RS H_2_SO_4_, (**e**) is RS KOH, and (**f**) is RS NaOH.

**Figure 5:**
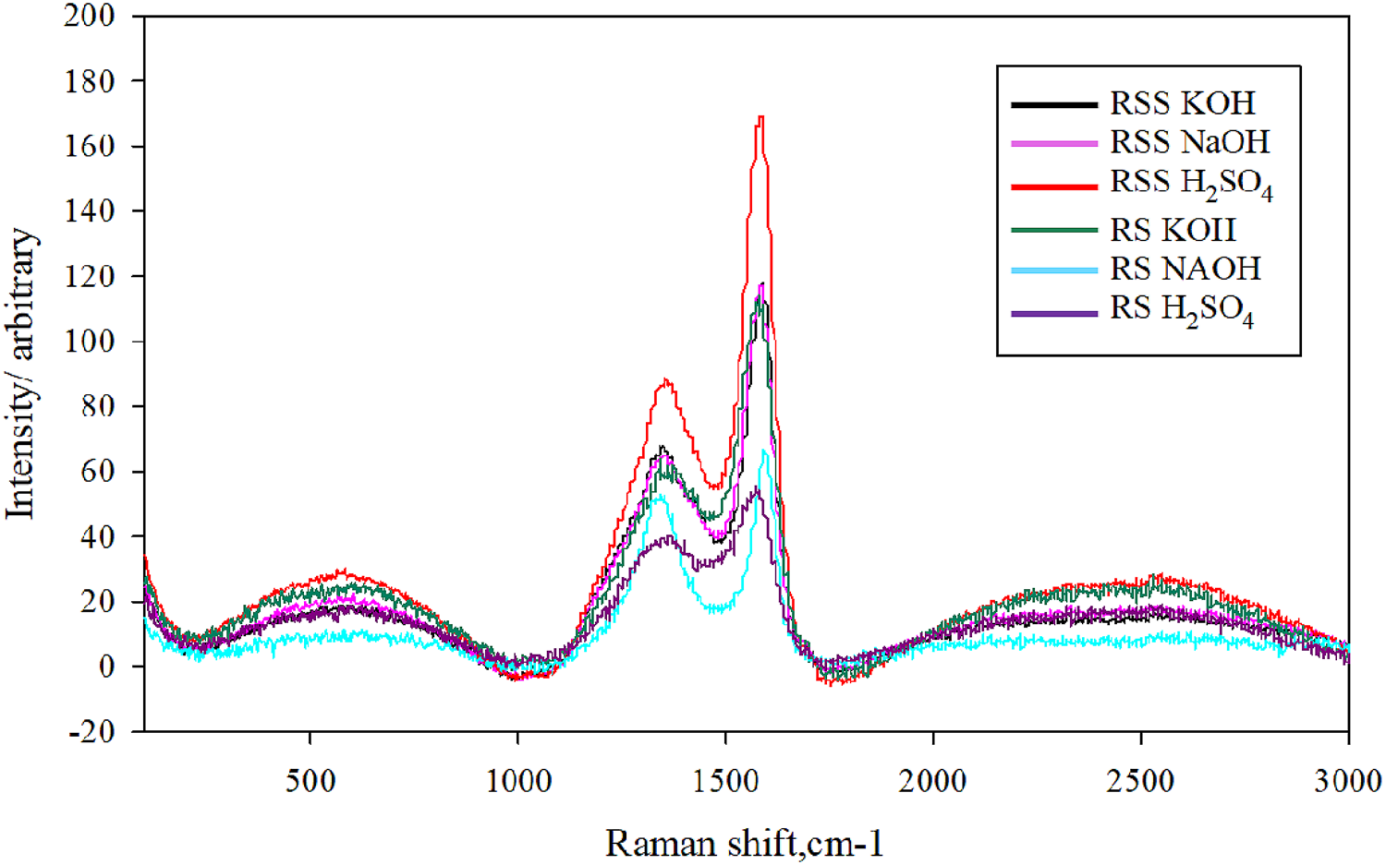
Raman spectroscopy of PAC using various activation solutions.

The cross-sectional morphology of the PACs is shown in Fig. 3a–f. They reveal the presence of a uniform-sized porous surface in the composites. The NaOH-activated PACs by RSS and NaOH by RS had the largest pores compared to the other. Based on the size of its pores and its morphology, the largest pores should be the most effective in the study of dye adsorption. Furthermore, as supported by the FTIR results, the Energy Dispersive x-Ray (EDX) mapping (Fig. 4a–f) showed a uniform distribution of the carbon element and oxygen compound. Figure 5 illustrates the Raman spectra of the PAC. The two extensive peaks in all plots of 1300–1400 cm^−1^ and 1550–1600 cm^−1^ refer to the D and G bands, respectively. The D band represents structural imperfections of oxygenated groups in the carbon atoms and a disordered sp3 carbon structure. In contrast, the G band showed sp2-ordered crystalline graphite-like structures that originated from activated carbons’ phonon models^52^. The ID/IG ratios indicated the size of the ring clusters in the sp2 and sp3 networks and the degree of oxidation in the PACs, which are close to one in the PAC^53–57^. Thus, the graphitization of the PAC was considered successful.

**Figure 6:**
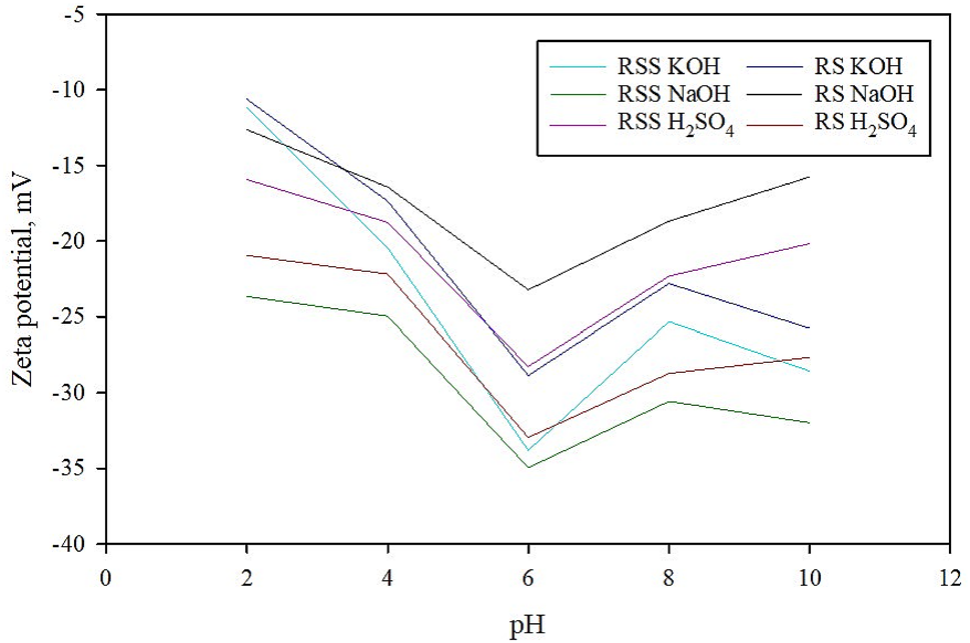
Zeta potential of the PAC adsorbent at various pH values.

Zeta potential was also conducted to analyze the magnitude of the charge repulsion/ attraction between particles used to measure the material’s stability (Fig. 6). The negative symbol in front of the zeta potential means that the scattering object’s net charge (including up to the slipping plane) was negative. MB uptake was high at alkaline solutions due to the electrostatic attraction between adsorbents and adsorbates’ surface. The PAC showed a significant decrease in charge at higher pH and reached its maximum negative charge at about pH 6 due to the PAC functional groups’ protonation. This demonstrated that the negatively charged PAC effectively removed the positively charged MB at the basic pH. This effectiveness is due to a more negatively charged surface, leading to a higher ionization rate and protonation of the adsorbent’s oxygen and hydroxyl groups.

### Initial adsorption performance

**Figure 7:**
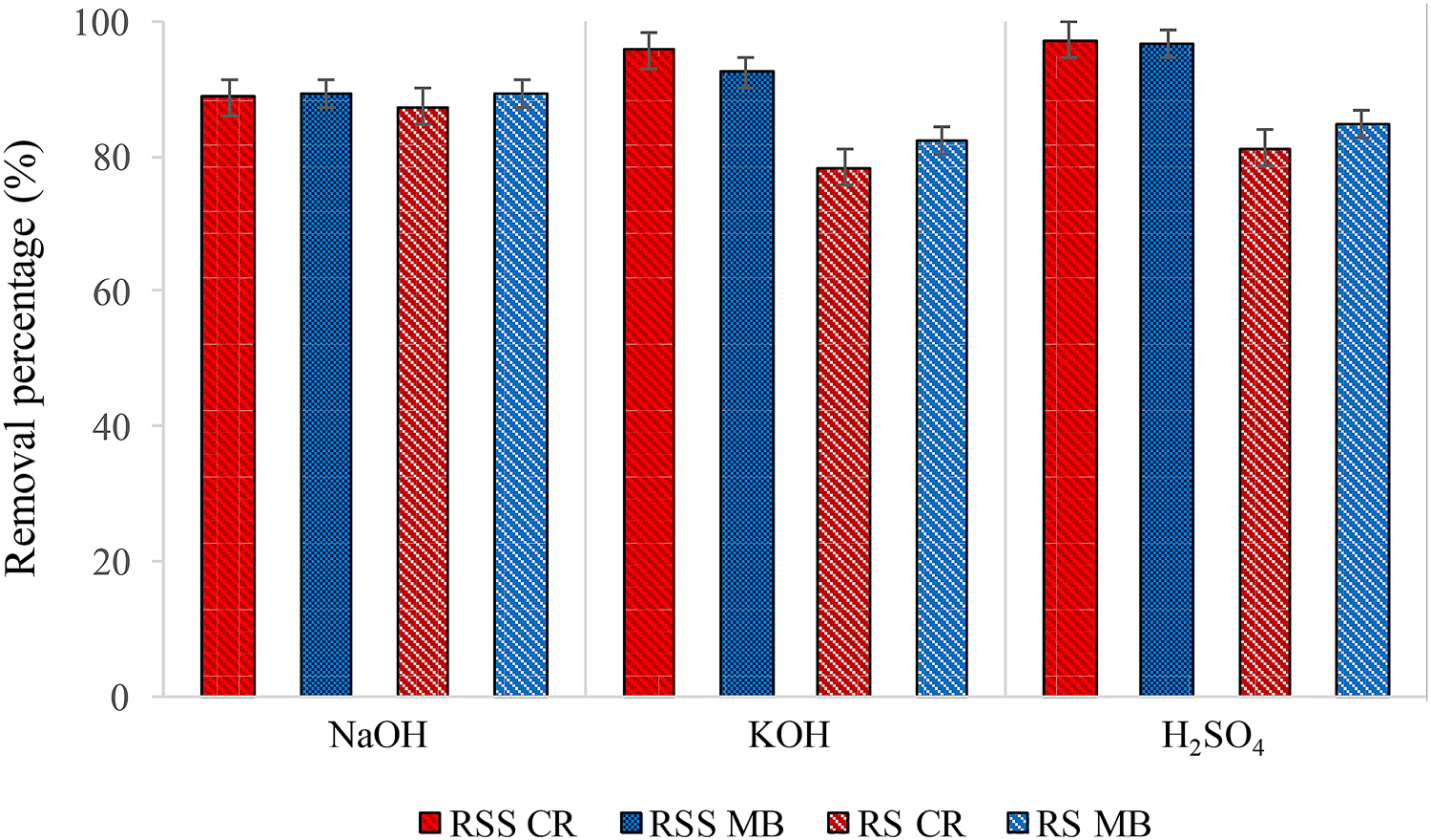
Adsorption removal study using various activating agents for MB and CR using PAC.

Figure 7 showed the removal percentage of dyes in the preliminary study. The FESEM supported this finding. EDX mapping results based on the pores’ size where the PACs impregnated with NaOH (RSS) and H_2_SO_4_ (RS) showed the highest percentage removal, therefore were used for the subsequent adsorption study. The existence of large functional groups proved in the FTIR analysis was also the primary reason for the adsorption capability.

## Adsorption study

### Adsorbent dosage

**Figure 8:**
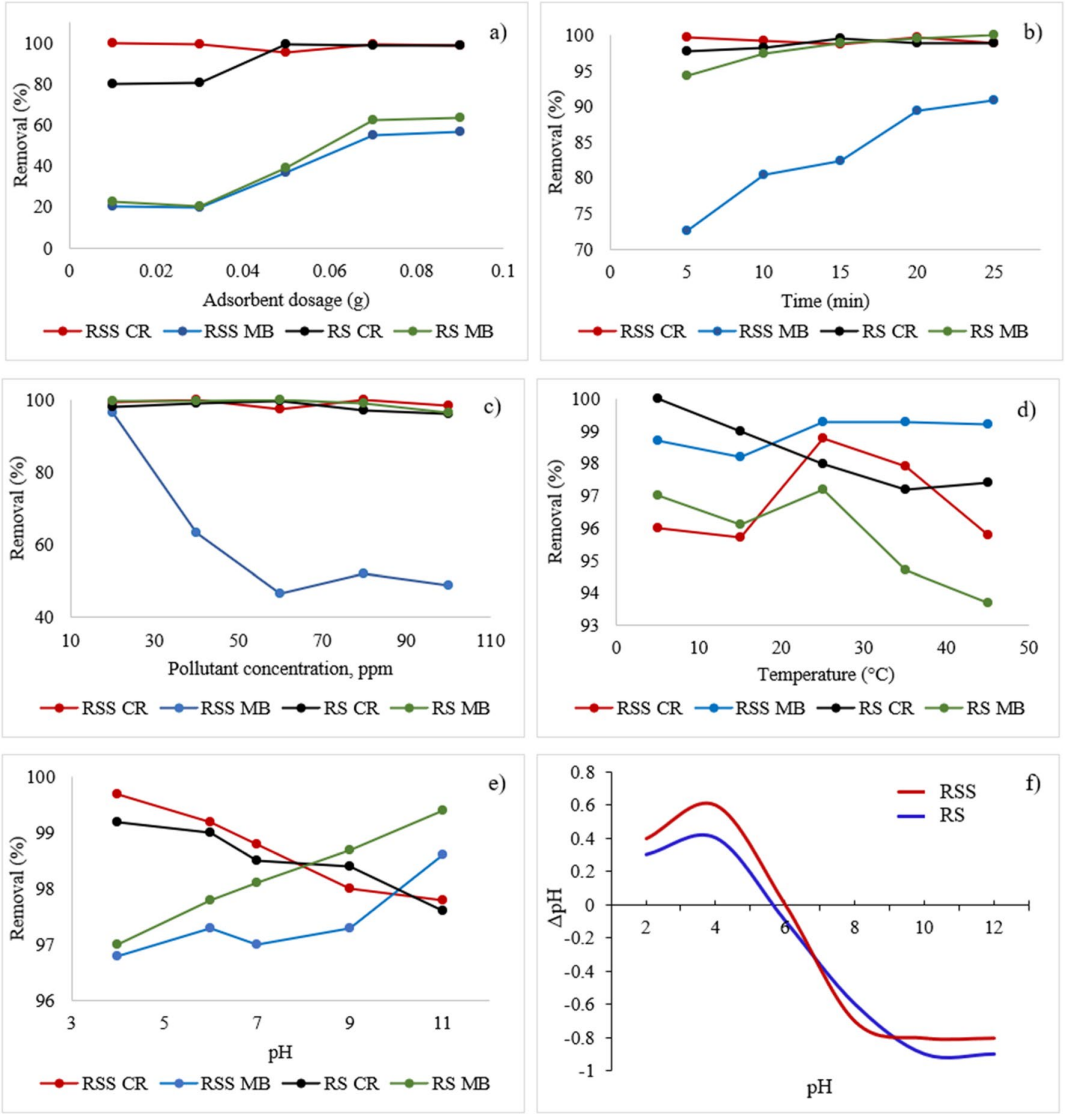
Effect of (**a**) adsorbent dose, (**b**) contact time, (**c**) pollutant concentration, (**d**) temperature, (**e**) pH on the adsorption of dyes by the PAC, and (**f**) The pHpzc values for RS and RSS.

The study of the adsorbent dosage is essential for avoiding waste of the material once the equilibrium phase has been reached^58^. The results obtained from the adsorbent dosage analysis for removing dyes using the PAC are shown in Fig. 8a. The percentage of MB removal increased from 21% to 59% when the PAC dosage increased from 0.01 g to 0.09 g. Generally, an increase in the adsorbent dosage implies more active sites or surface area for the interaction between the dyes and PAC. The availability increases the percentage of the dyes removed^58,59^. For CR, the adsorbents performed well, even with a low adsorbent dosage, indicating that low CR has enough active sites for the CR to react and adsorb effectively. At the adsorbent dose range of 0.05-0.07, the adsorption efficiency did not change significantly for both dyes, and the resulting graph is flat. This resulted because the number of active sites adsorbent in the aqueous solution for the dye molecules was greater than the number of dye molecules. As a result, several active sites were unutilized^31,33^. Hence, the reduction in adsorption capacity was observed.

### Contact time

Figure 8b shows the effect of contact time on the adsorption of dyes. Figure 8b shows that the increase in contact time led to a slight rise in CR’s adsorption performance. However, the removal percentage was extremely high, ranging from 98% to 99%, demonstrating that CR’s initial adsorption rate by the PAC (RSS and RS) was extremely fast in removing CR. A large number of active sites of the functional adsorbent groups contributed to higher adsorption rates for CR, nearly 99% of removal.

As for MB (RSS), the adsorption performance increased over time. In the beginning stage of the adsorption process, the adsorption rates were typically high due to the improved collision of dye molecules in aqueous solutions. When it reached a state of equilibrium in which a reasonable amount of dye was adsorbed, the molecules’ collisions were lower due to fewer active sites. At this point, the adsorption rate was slow and constant but steadily increasing.

### Dye concentration

Figure 8c shows the removal percentage for both CR and MB decreased as the dye concentration increased. The decline in the uptake percentage of dyes is due to the excessive number of pollutants that exceeded the number of available adsorption sites^48,60^. The PAC showed the best uptake capacity for removing CR and MB at concentrations of 40 ppm and 20 ppm, respectively, for both RSS and RS. The results showed that the PAC adsorbent possessed a specific number of active sites for the CR and MB dyes’ adsorption. This indicated many unused active sites due to the greater number of active sites for the dye molecules’ adsorbent than the number of dye molecules. This led to a reduction of adsorption capacity.

### Temperature

Another vital parameter that directly impacted the adsorption of dyes was temperature, affecting the solid-solute interface and mobility of pollutants during adsorption^61^. In this study, the efficiency of both MB and CR adsorptions decreased with increasing solution temperature (Fig. 8d). This situation may be due to shrinkage and alteration of active sites on the adsorbent at a higher temperature, reducing the active adsorbent surface, and leaching the dyes into the solution due to a higher separation between the dye molecules and the adsorbent at high temperature^31–33^. This phenomenon demonstrated that both MB and CR’s adsorption is an exothermic process that favors low-temperature solutions.

### pH

The effect of pH on dye adsorption affects the interaction between the PAC and the dyes. The pH of the solution influences the surface charge of the adsorbent and the ionization of the adsorbate. However, hydrophobic interaction, hydrogen bonds, π-π interaction, and n-π interaction also affect the adsorption performance in aqueous solutions^58,62,63^. The effects of pH are presented in Fig. 8e.

The highest rate of removal of CR was at pH 4, with an uptake of almost 100%. CR is negatively charged and effective when removed by the PACs at pH 4. The attraction forces between an aqueous solution with low pH (high value of H^+^ charge) and the negatively charge CR dye is high, causing the adsorption efficiency to increase^31,33^. Similar results were also reported for the adsorption of CR in several studies^1,2,64,65^. According to Barkauskas et al.^66^ and Debnath et al.^61^, CR uses two mechanisms in the process of adsorption: π-π interaction between the basal planes of the PAC and aromatic rings of the dyes. Also present are hydrogen bonds between oxygen and the amino groups of CR, oxygen, and the hydroxyl groups of the adsorbent. The condition with the lowest point of the surface charge of the adsorbent (pH 4) allows the ionization and protonation of the adsorbent’s functional groups to reach their maximum rate, contributing to the highest adsorption uptake of CR. The MB dye showed excellent adsorption in a basic medium, as expected due to its cationic properties. As the pH increased, the H+ charges in the solution decreased, resulting in less competition between positive ions and dye molecules. Because the PAC was negatively charged at high pH, an increase in the electrostatic force of gravity between the surface of the adsorbent and cationic dye molecule exists, leading to increasing adsorption efficiency. The pHpzc values for RS and RSS were 5.6 and 6.0, respectively (Fig. 8f). This indicated that when pH<pHpzc, the adsorbent surface is positively charged and tends to absorb anionic species or negatively charged dye, which in this case is CR, while cationic species (MB) tends to absorb on the surface of negatively charged adsorbents (pH>pHpzc) due to electrostatic interactions^31^.

### Adsorption kinetics

As shown in Table 1, the correlation coefficient of determination (R^2^) of the pseudo-second-order kinetic model exceeded 0.99 (RSS and RS), significantly higher than those of the pseudo-first-order kinetic model. This finding indicated that a chemical effect was involved in the adsorption of dyes on PAC. Moreover, the calculated values of the equilibrium adsorption capacity (q_e_) from the pseudo-second-order showed an absolute agreement with the experimental values (q_e,exp_). These results further indicated that the pseudo-second-order model was more suitable for the adsorption of dye onto the PAC, showing that the adsorption was chemically controlled. In addition, the lower correlation coefficient values in the pseudo-first-order and the Elovich model showed that these models did not agree on how to explain the adsorption mechanism, suggesting that the process was indeed efficient and controlled by chemical adsorption, as mentioned earlier^67–72^. This process involved the exchange of electrons between the adsorbates and adsorbent^31,47,73–76^. The α parameter values using Elovich kinetic model for RSS for both dyes were higher than RS, indicating that RSS had a greater tendency and ability to adsorb dyes. The experimental results also supported these findings. The values for the intraparticle diffusion rate constant (K_ID_) for RSS in the adsorption process of both dyes was higher than RS, also indicating that RSS had better adsorption and more improved bonding between the adsorbent and adsorbate particles^31–34,68,70–72^.

**Table 1:** The correlation coefficient, R^2^ of pseudo-first and pseudo-second orders of RS and RSS.

Kinetic model	Parameter	CR		MB	
		RSS	RS	RSS	RS
Pseudo-first-order	R^2^	0.89	0.95	0.98	0.98
	q_e_ (mg/g)	19.47	1.97	19.86	11.55
	k_1_ (min^-1^)	0.03	0.35	0.05	0.45
Pseudo-second order	R^2^	0.99	1.00	1.00	1.00
	q_e_ (mg/g)	24.65	21.93	25.43	20.65
	k_2_ (g mg^-1^ min^-1^)	0.02	0.2212	0.03	0.1755
Intraparticle diffusion	K_ID_	1.42	0.81	1.64	0.78
	C	1.87	2.77	1.54	2.39
	R^2^	0.91	0.96	0.89	0.88
Elovich	α (mg/g.min)	1.96	1.07	1.48	0.63
	β (g/mg)	0.73	2.89	0.51	11.84
	R^2^	0.85	0.73	0.81	0.94

### Adsorption isotherms

Isotherm models of Langmuir, Freundlich, and Dubinin-Radushkevich were fitted to the experimental data; the parameters obtained for these equations are tabulated in Table 2. According to the values of R^2^ and other parameters in Table 2, the fittings of Langmuir, Freundlich, and Dubinin- Radushkevich models showed the degree of surface saturation by using isotherm models. The values of *E* for the PACs adsorption process were 1.65 kJ/mol (RSS) and 2.34 kJ/mol (RS) for CR dye and 1.84 kJ/mol (RSS) and 2.67 kJ/mol (RS) for MB, demonstrating that the adsorption process of both dyes was desirable and physical^31^. Adsorption of CR by PAC was well-fitted with the Langmuir isotherm. This verifies that the adsorption occurred in a monolayer manner^68–72^ because the PACs’ large surface area influenced the adsorption of large CR molecules onto the PACs^48^. The value of R^2^ determines the models’ suitability, and the Q_max_ value was calculated to be considerably high, as shown in Table 2. On the other hand, the Freundlich model was determined to be suitable to explain the adsorption of MB, considered the amount of CR adsorbed per unit mass of PACs used. According to these results, the PAC’s adsorption of the dyes occurred mainly in monolayer and multilayer manners. The monolayer adsorption was determined by the Langmuir model, while the Freundlich model showed the adsorption of the dyes on the surface of the adsorbent in multilayers. The presence of functional groups of PACs which contributed to π-π interaction and electrostatic interaction between the adsorbents and adsorbates explained the adsorption mechanism^31,48,74–78^.

**Table 2:** Parameter values of the isotherm models in the adsorption of dyes by the PAC adsorbent.

Models	Parameter	CR		MB	
		RSS	RS	RSS	RS
Freundlich	k (mg g^−1^)	0.57	0.32	0.48	0.28
	1/n	0.46	0.53	2.14	0.18
	R^2^	0.89	0.67	0.76	0.91
Langmuir	R^1^	0.99	0.79	0.79	0.51
	Q_max_ (mg g^−1^)	659.35	227.27	458.43	769.23
	R^2^	0.98	0.51	0.64	0.87
Dubinin-Radushkevich	Q_max_ (mg g^−1^)	471.77	93.61	479.81	63.37
	E (kJ/mol)	1.65	2.34	1.84	2.67
	R^2^	0.81	0.76	0.67	0.54

### Thermodynamics study

A positive ΔG generally showed that an external source of energy is required during the adsorption process. Meanwhile, a negative ΔG factor indicated the feasibility of the adsorption process and its spontaneous nature without the need for an external energy source. The negative ΔG values obtained in this study (Table 3) showed that the adsorption process that removed MB and CR occurred spontaneously without the need for an external energy source. In addition, the ΔG values for CR were lower than -1 kJ mol^-1^ at various temperatures, which showed that physical adsorption is an effective mechanism in this process^47,67^. In this study, the values of ΔH for the CR and MB were -0.099 kJ mol^-1^ and -1.158 kJ mol^-1^, respectively, confirming the adsorption process was exothermic^31,33^. The negative value in both MB and CR for ΔS was ascribed to the fact that the adsorbates lose randomness in the solid-solute interface during the adsorption process^47,58,79–81^. However, the ΔG values’ temperature was not significant within the range of temperatures investigated. The results of this analysis clearly showed that the adsorption of both MB and CR were exothermic and spontaneous. The process contributed to reduced adsorption capability as temperature increased (Fig. 8e).

**Table 3:** Thermodynamic parameters in the adsorption of MB and CR by the PAC adsorbent.

Sample	Temperature (K°)	ΔH (kJ mol^−1^)		ΔS (kJ mol^−1^)		ΔG (kJ mol^−1^)	
		CR	MB	CR	MB	CR	MB
RSS	298	− 0.099	− 1.158	− 0.017	− 0.063	− 0.094	− 1.131
	303					− 0.093	− 1.129
	313					− 0.091	− 1.122
	323					− 0.090	− 1.117
RS	298	− 0.097	− 1.135	− 0.042	− 0.008	− 0.092	− 1.119
	303					− 0.091	− 1.116
	313					− 0.089	− 1.115
	323					− 0.088	− 1.111

### Effects of ionic strength, PAC regeneration, and comparison with other adsorbents

**Figure 9:**
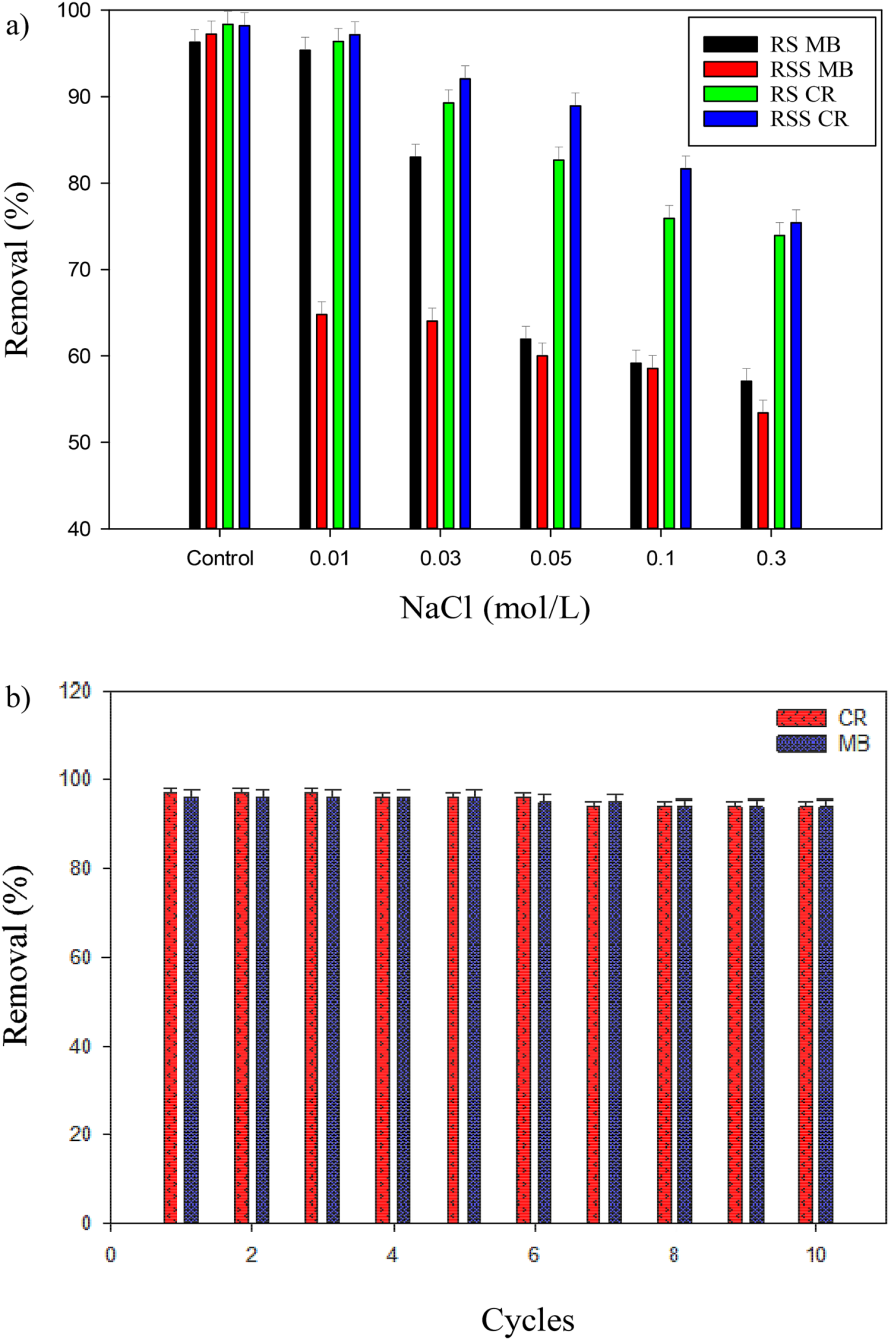
(**a**) Influence of the solution ionic strength on dyes adsorption capacity; and (**b**) Regeneration ability of PAC for removing MB and CR dyes.

The effects of ionic strength in an aqueous solution were studied using a NaCl solution (0.01–0.3 mol/L) as the electrolyte solution to examine the adsorption process’s efficiency on RS and RSS activated carbon. The results are shown in Fig. 9a. At a higher concentration of the electrolyte solution, the dye removal efficiency using RS and RSS activated carbon decreased. This activation was due to the competition between the Na^+^ and PAC molecules. At a higher NaCl solution concentration, the negatively charged functional groups (OH^-^, COO^-^) interacted electrostatically with Na^+^ ions^33^. The same charges on the adsorbent surface between the two created a repulsive electrostatic force, causing the adsorption process’s efficiency to decrease.

In this study, the regeneration was accomplished through ten cycles. PAC adsorbents were washed with HCl in between cycles, and the results are presented in Fig. 9b. The adsorption percentage was reduced to only 1% after six times recycling the adsorbent in CR removal, and there was no decline was observed in MB removal. However, the change in removal efficiency was seen in the seventh cycle, with a 3% decline in the CR and a 2% decline for MB removal performance. The adsorbents’ excellent life cycle and regeneration ability could be correlated to the pretreatment methods and high porosity of the synthesized adsorbent.

Table 4 shows the data from previous studies using different types of biomass-based adsorbents. By comparison, adsorption of MB and CR using PAC derived from RSS and RS showed encouraging results compared to other adsorbents previously used. The highest value reported was 612.1 mg g^-1^ of MB removed using activated carbon derived from date palm seeds^76^. In this study, the highest adsorption capacity was 769.23 mg g^-1^ for MB using rubber seeds, while CR was 458.43 mg g^-1^. Therefore, rubber seeds appeared to be the most effective in removing MB, while the shells are the best option to remove CR. Due to the synergistic effect of various functional groups, biomass-derived PACs demonstrated the superior adsorption capability for MB and CR.

**Table 4:** Dye adsorption capacities of the resulting activated carbons as reported in the literature.

Adsorbent	Adsorbate	Q_max_ (mg g^−1^)	Reference
Methylene blue	Date stones	381.79	Ahmed and Theydan^40^
Methylene blue	Oil palm fibers	382.32	Foo and Hameed^41^
Methylene blue	Coconut husks	418.15	Foo and Hameed^42^
Methylene blue	Durian shells	410.85	Foo and Hameed^43^
Methylene blue	Date pits	244.00	Ashour^81^
Methylene blue	Date pits	345.00	Reddy et al.^82^
Methylene blue	Date stones	316.11	Foo and Hameed^83^
Methylene blue	Date palm seeds	612.10	Islam et al.^84^
Methylene blue	Date pits	455.00	Mahmoudi et al.^85^
Methylene blue	Date palm rachis	198.80	Daoud et al.^86^
Methylene blue	Microalgal biomass	113.00	Yu et al.^87^
Methylene blue	Coconut shell	156.25	Yağmur et al.^88^
Methylene blue	*N. diderrichii*	35.09	Omorogie et al.^89^
Methylene blue	Banana roots	165.00	Paluri et al.^90^
Methylene blue	Ackee apple pods	47.17	Bello et al.^91^
Methylene blue	Soya wastes	90.00	Batool and Valiyaveettil^92^
Methylene blue	Corncob wastes	636.94	Zhou et al.^93^
Congo red	Watermelon rind	17.00	Masoudian et al.^2^
Congo red	Bael shells	98.03	Ahmad and Kumar^63^
Congo red	Grape wastes	455.00	Sayğılı and Güzel^64^
Congo red	Microalgal biomass	164.35	Yu et al.^87^
Congo red	Speargrass leaves	313.00	Bello and Banjo^94^
Congo red	Date pits	105.00	Belhachemi and Addoun^95^
Congo red	Peanut shells	150.00	Lawal et al.^96^
Congo red	Coffee wastes	90.90	Lafi et al.^97^
Congo red	Wood wastes	8.00	Stjepanovic et al.^98^
Congo red	Rubber seed shells	**458.43**	Present study
Methylene blue	Rubber seed shells	**659.35**	Present study
Congo red	Rubber seeds	**227.27**	Present study
Methylene blue	Rubber seeds	**769.23**	Present study

## Conclusions

In this study, PAC adsorbents from RS and RSS were successfully synthesized. The results of FESEM and EDX mapping showed a porous surface in uniform sizes, with PAC activated by NaOH (RSS) and H_2_SO_4_ (RS) having the largest pores. The prepared adsorbent showed excellent CR and MB adsorptions. The affinity for adsorption by the PACs was due to its single-layered planes with the functional groups (hydroxyl, alkoxy, and carboxyl), as reported in FTIR analysis. The π−π interactions and hydrogen bonds of the PACs with CR and MB enhanced the adsorbent performance. Based on multiple studies, the PACs activated by NaOH were more effective than the other two impregnation agents (NaOH and H_2_SO_4_) for RSS, and H_2_SO_4_ was found to be the best for RS. The maximum adsorption capacities of the PACs were 458.43 mg g^-1^ (CR by RSS), 659.35 mg g^-1^ (MB by RSS), 227.27 mg g^-1^ (CR by RS), 769.23 mg g^-1^ (MB by RS). The kinetic and isothermal data were well-suited to the pseudo-second-order kinetic model. Based on the isotherm models, more than one mechanism was involved in the adsorption process, a monolayer for the anionic dye and a multilayer for the cationic dye.

A thermodynamic study showed that both dyes’ adsorption process was spontaneous and exothermic due to the negative values of the enthalpy (ΔH) and Gibbs free energy (ΔG). An acidic pH range and lower temperature of the solution were unfavorable. At a higher concentration of the electrolyte solution (NaCl), the dye adsorption efficiency using RS and RSS activated carbon decreased due to the competition between the Na^+^ and PAC molecules. Lastly, the adsorbents’ superior life cycle and regeneration ability with only a 3% decline for CR and a 2% decline for MB could be correlated to the pretreatment methods and high porosity of the synthesized adsorbent.

In this study, the RSS-derived PACs were the most suitable for removing CR dyes, and RS-derived PACs were the best option to remove MB dyes. The results demonstrated that the PAC is a promising adsorbent for purifying and treating dyes in discharged wastewater, particularly in the industrial sector. Analysis and experiments on the effectiveness of other local biomass wastes and the potential for the upscaling of the synthesized PACs into increased production should be explored to solve real-world water quality challenges. In addition, the total energy required to scale up from laboratory tests to a full-scale commercial production plant is worth further investigation to produce environmentally sound technologies for wastewater treatment.

## Methods

### Materials

All chemicals used in this study were analytical grade. Potassium hydroxide (KOH), sodium hydroxide (NaOH), sulfuric acid (H_2_SO_4_), and hydrochloric acid (HCl) were obtained from Acco Lab Supplies, Malaysia. The MB and CR dyes were purchased from Sigma-Aldrich, Malaysia.

### Preparation of PAC

**Figure 10:**
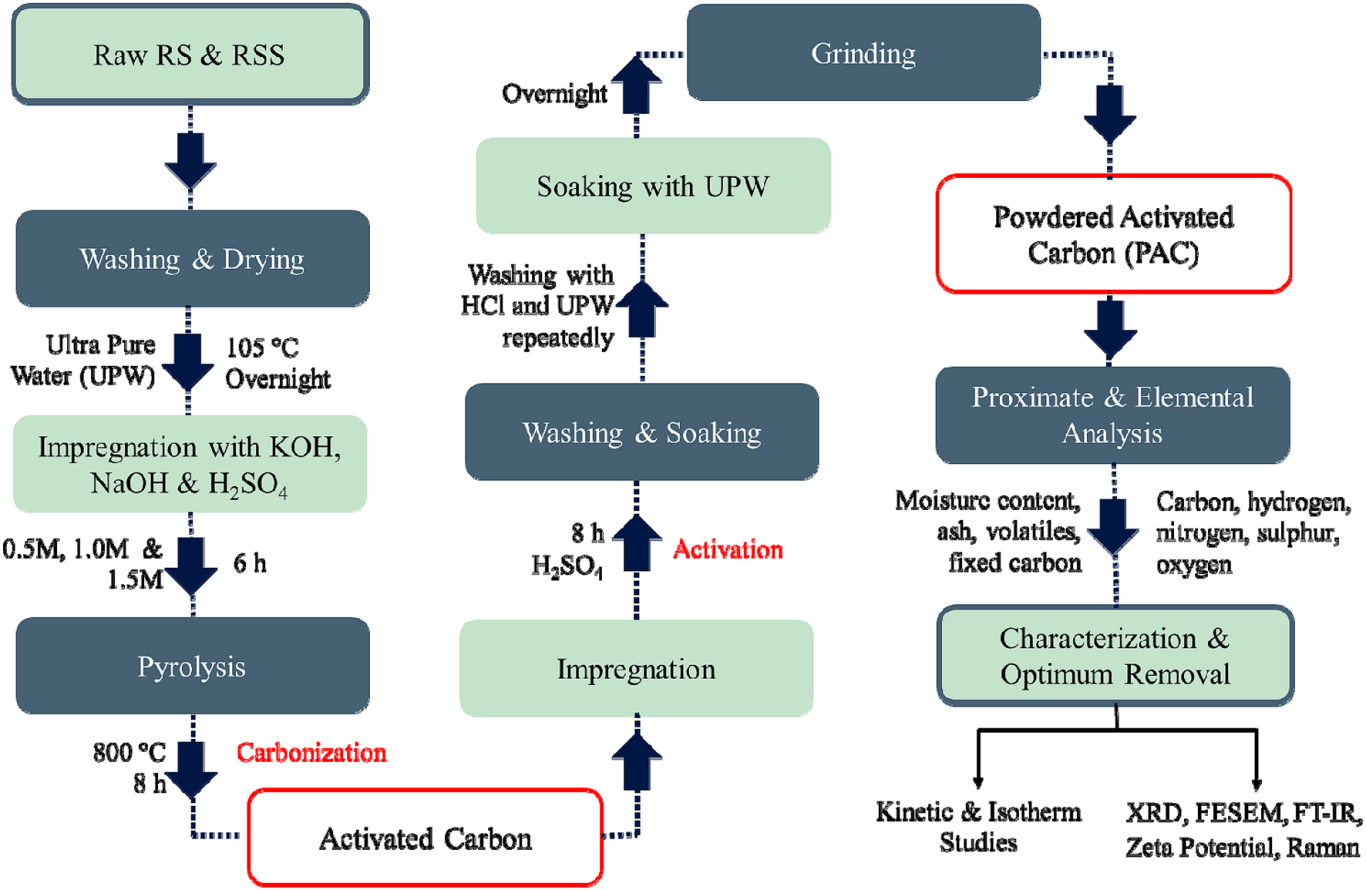
Process for fabrication and synthesis of PAC from RS and RSS.

The RS and RSS were washed thoroughly to remove any dirt and impurities and then dried overnight in an oven at 105°C. Acids and base solutions, 0.5 M KOH, 1.5 M NaOH, and 1.5 M H_2_SO_4,_ were prepared. The dried sample was cooled at room temperature before proceeding to the next step. The sample was then impregnated with prepared solutions of three different concentrations for six hours. One beaker of dried RSS was soaked with ultra-pure water to be kept as control. After six hours, the impregnated sample was pyrolyzed and gradually carbonized in a furnace for eight hours at 800°C. The pyrolyzed sample was then cooled at room temperature and soaked with H_2_SO_4_ for eight hours to be activated. The sample was then washed repeatedly with 0.1 M hydrochloric acid (HCl) and ultra-pure water until no color or residual substances remained. For further washing, the sample was soaked with ultra-pure water overnight to ensure it was clear of any impurities, acids, or bases. The processes of preparing and synthesizing the RS and RSS into the PAC are shown in Fig. 10.

### Characterization

An FTIR analysis was conducted using a Nicolet 6700 Thermo Scientific-FTIR 12 spectrometer (United States). The purpose was to identify the exterior functional groups and chemical bonds of the synthesized PAC. XRD using a Bruker AXS D8 Advance Karlsruhe (Germany) and XRD DIFFRAC EVA software were used to examine the adsorbent structure, composition, and material properties. Morphological surface mapping of the PAC adsorbent was conducted using a Gemini model 18 SUPRA 55VP-ZEISS Oberkochen (Germany) FESEM equipped with energy-dispersive X-ray 19 spectroscopy (EDX). Zeta potential analysis was used to determine the PAC’s surface charge in a colloidal solution using a Zeta Sizer (Nano-ZS, Malvern 17 Instruments Inc. UK). Raman imaging spectroscopy analysis was also conducted (Thermo Scientific I Model: DXR2xi) to analyze the PAC’s chemical structure, crystallinity, phase, and polymorphic and molecular interactions based on the interaction of light within the adsorbent. The pHpzc (pH of point zero charge) was also measured to determine the electrostatic nature of the adsorbent’s surface.

### Adsorption study

The effect of adsorbent dosage, pollutant concentration, solution pH, contact time, and temperature was measured and compared with the accuracy of three replicates. The flow and adsorption parameters are shown in Table 5. For the initial adsorption, 10 ml of synthetic CR and MB solutions were used with different impregnation mediums (KOH, NaOH, and H_2_SO_4_) of the PAC adsorbent. Equal amounts of adsorbent, 0.01 g, were added, and the contact time of the adsorption process was set at two minutes under room temperature. The impregnated PAC’s best removal efficiency was identified based on the synthetic solutions’ concentration, labeled A, and used in all of the experiments that followed. The following experiments were conducted again but with different adsorbent dosages ranging from 0.02 g to 0.08 g. Concentrations of synthetic solutions recorded as efficient for removal were labeled B and used in the following steps. The next steps were repeated with different contact times, pollutant concentrations, solution pH, and temperature and labeled C, D, E, and F, consecutively (Table 5). The solution’s pH was regulated to the desired values using NaOH and HCl solution (0.1M, pH: 2–10).

**Table 5:**
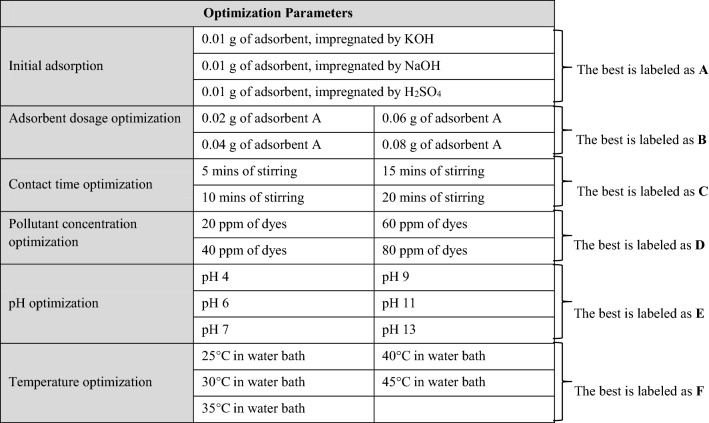
Optimization of the adsorption parameters.

The removal percentages of CR and MB by the PAC adsorbents were calculated. The solutions’ concentration was determined using a UV DR 3900 (HACH, USA) at wavelengths of 496 nm for CR and 668 nm for MB. The uptake capacities of the adsorbates by the PAC adsorbent were calculated using Eq. (1):

1 $$q_{e} = \frac{{\left( {C_{0} - C_{e} } \right)V}}{M}$$

where q% is the overall removal percentage, q_e_ (mg g^-1^) is the adsorption capacity, C_0_ (mg l^-1^) is the initial pollutant concentration, C_e_ (mg l^-1^) is the pollutant concentration after the adsorption process, V (l) is the solution volume, and M (g) is the mass of the adsorbent.

### Isotherm model analysis

Adsorption isothermal models were analytical and mathematical patterns based on the Langmuir, Freundlich, and Dubinin-Radushkevich models. Using those models, equilibrium was plotted as the solid phase (q_e_) versus the liquid phase concentration (C_e_). The models explained the diffusion and the concentration of pollution^60,67^. These data are essential to understand the uptake mechanisms and the interaction between adsorbate and the adsorbents. The results indicated that the removal of dyes by the PAC involved multilayer adsorption uptake on a heterogeneous surface of adsorbate; the amount of adsorbate adsorbed increased infinitely with an increase in concentration^99^.

The Langmuir model, with a linear-plotted 1/q_e_ versus 1/C, was determined using Eq. (2):

2 $$\frac{1}{{q_{e} }} = \frac{1}{{q_{m} }} + \frac{1}{{K_{L} q_{m} C_{e} }}$$

where q_e_ is the equilibrium adsorbate concentration in solution, q_m_ is the maximum adsorption capacity, and KL is the Langmuir constant in L mg^-1^. The Langmuir adsorption isotherm model describes the adsorption sites’ homogenous surface and the absence of interactions between the adsorbate molecules. The Freundlich model with linear-plotted log q_e_ versus log C_e_ is shown in Eq. (3):

3 $$\log q_{e} = \log K_{f} + \frac{1}{n}\log C_{e}$$

where K_f_ is an indicator of the adsorption capacity, and 1/n is the adsorption intensity. A linear form of the Freundlich expression yielded the constants K_f_ and 1/n.

The Dubinin-Radushkevich isotherm model was conducted to examine the biosorption process’s energy in detail, whether the process is physical or chemical. The linearized form of the Dubinin-Radushkevich equation is shown in Eq. (4)^31^.

4 $$\ln q_{e} = \ln q_{m} + \beta \varepsilon^{2}$$

### Kinetic model analysis

An analysis was conducted to determine the adsorption kinetics of dyes onto the PAC. The adsorption kinetics for these adsorbents were determined based on the pseudo-first-order, pseudo-second-order, Elovich models, and intraparticle kinetic models. These models were used to analyze the PAC’s dye removal process. The adsorption mechanism was chemically explained as the probability of adsorption properties, which typically involves monolayer adsorption due to its specific bonding between adsorbates and the surface of the adsorbent^99^. Eq. (5) shows the calculation of the pseudo-first-order model:

5 $$\ln \left( {q_{e} - q_{t} } \right) = \ln \left( {q_{e} } \right) - k_{1} t$$

where q_e_ (mg g^-1^) and q_t_ (mg g^-1^) are the amounts of dye removal or adsorbate adsorbed at equilibrium and at time t; k_1_ is the rate constant of this first-order model. The pseudo-second-order equation is written in linear form as follows (Eq. (6))^60^.

6 $$\frac{1}{{q_{t} }} = \frac{1}{{k_{2} q_{e}^{2} }} + \frac{1}{{q_{e} }}t$$

where the slope and intercept of (t/q_t_) versus t were used to calculate the pseudo-second-order rate constant (k_2_) and adsorbate adsorbed at equilibrium (q_e_).

The pseudo-second-order kinetic (Elovich equation) assumed that the real media surfaces were heterogeneous based on their energy. The linear equation is shown in Eq. (7):

7 $$q_{t} = k_{i} t^{0.5} + c$$

where k_i_ is the intraparticle diffusion constant, and c is the intercept. The pseudo-second-order model showed the best fit to the experimental data with the highest squared correlation coefficients of determination (R^2^ = 0.999)^100^.

### Thermodynamics study

To calculate the thermodynamic factors that can provide a better understanding of the nature of the adsorption reaction and its feasibility, a Gibbs free energy change (ΔG), entropy change (ΔS), and enthalpy change (ΔH) are required. All of the parameters were calculated at different temperatures, according to Eqs. (8) and (9). T (°K) is the absolute temperature, K_L_ is the equilibrium constant, and R (8.314 J mol K^-1^) is the universal gas constant. The ΔH, ΔS, and ΔG values were calculated based on the slope and y-intercept of Eq. (10) for both MB and CR:

8 $${\Delta G} = { } - {\text{ RT ln }}K_{L}$$

9 $$\Delta G = \Delta H - T \Delta S$$

10 $$\ln K = \frac{{\Delta S}}{R} - \frac{{\Delta H}}{{{\text{RT}}}}$$

### Ionic strength and regeneration of the PAC

The active sites of the adsorbent surface had ions that affected the adsorption process. To study the stability of the dyes and the adsorbents, the ionic intensity test was applied using NaCl solution as the electrolyte solution in various concentrations, which are 0.01, 0.03, 0.05, 0.1, and 0.3 mol/L.

The adsorbent’s ability to regenerate and reuse without significant changes in the adsorption capacity and efficiency played a vital role in reducing the cost of the adsorption process and synthesized adsorbent. To test the reusability and regeneration capability of the material, ten cycles of the adsorption process were conducted at optimum adsorption conditions. Adsorbents were recovered using a centrifuge (Sigma 6-16S, Germany, 8000 rpm, 10 min). After each cycle, the adsorbents were washed and regenerated using 1% HCl solution. The regenerated adsorbents were dried in an oven at 60 °C before each cycle in the adsorption process.
